# Social support, social constraint, and psychological adjustment in patients with colorectal cancer

**DOI:** 10.1007/s10865-025-00565-y

**Published:** 2025-04-01

**Authors:** Nirvi B. Ajmera, Brian D. Doss, Youngmee Kim

**Affiliations:** Department of Psychology, University of Miami, Miami, FL USA

**Keywords:** Cancer, Psychological adjustment, Social support, Social constraint, Romantic partner

## Abstract

Cancer patients’ social networks, particularly their spouses or romantic partners, can promote or undermine their psychological adjustment. This study examined the relative associations of partner social support and social constraint with patients’ psychological adjustment and further tested gender’s moderating role in these associations. Participants were 124 patients newly diagnosed with colorectal cancer (*M *age = 56.6 years, 34% female), who completed questionnaires on perceived spousal social support and social constraint, depressive symptoms, and life satisfaction. Findings revealed that greater social constraint was significantly associated with lower life satisfaction regardless of gender; however, greater social constraint was only associated with greater depressive symptoms in male patients. No significant associations or interactions with social support were found. Findings highlight the importance for patients—especially male patients—with cancer to feel able to disclose cancer-related thoughts and feelings to their partners and call for more consistent operationalization and measurement when studying patients’ social functioning.

## Introduction

As all-site cancer survival rates continue to improve (American Cancer Society, 2024), the psychological adjustment of patients with cancer has gained increasing attention (De Ridder, Geenan, Kuijer et al., 2008). Patients with cancer experience depressive symptoms at rates two to three times higher than the general population (Caruso et al., 2017; Hartung et al., 2017; Massie, 2004; Watts et al., 2014), with 8–24% of patients with cancer reporting depressive symptoms that meet clinical criteria (Krebber et al., 2014; Mitchell et al., 2011; Pilevarzadeh et al., 2019). Elevated depressive symptoms in patients with cancer include and have been associated with greater fatigue, sleep disturbance, weight loss, decreased energy, psychomotor changes (Barrera & Spiegel, 2014; Smith, 2015), suicidal ideation, poor quality of life (Chochinov, 2001; Smith, 2015), poor treatment adherence (DiMatteo & Haskard-Zolnierek, 2011; Manning & Bettencourt, 2011; Theofilou & Panagiotaki, 2012), cancer recurrence (Wang et al., 2020), and greater mortality (Pinquart & Duberstein, 2010; Satin et al., 2009; Wang et al., 2020).

Patients with cancer have also reported levels of life satisfaction lower than (Vázquez et al., 2015) or on par with (Büssing et al., 2009; Ellis et al., 2019; Tsai et al., 2023) those of healthy populations. Higher levels of life satisfaction in patients with cancer have been associated with better quality of life, less severe physical symptoms, greater acceptance of illness (Polański, Chabowski, Świątoniowska-Lonc et al., 2020), and lower pain intensity (Dezutter et al., 2017). Overall, understanding the factors that are associated with patient depressive symptoms and life satisfaction are critical to best support patient wellbeing and physical health.

Patients’ interpersonal context is one such factor and significantly correlates with depressive symptoms and life satisfaction. According to the social cognitive processing (SCP) model, individuals in patients’ social networks may promote or disrupt patients’ emotional and cognitive processing of potentially traumatic experiences such as cancer diagnosis and treatment (Lepore, 2001; Sippel, Pietrzak, Charney et al., 2015). Social support, defined in this study as patients’ perceptions of support received from individuals in their social network (Haber et al., 2007; Luszczynska et al., 2013), promotes such emotional and cognitive processing. For example, individuals from patients’ support networks may provide emotional comfort and offer new perspectives that allow patients to process, habituate to, and reappraise their cancer-related experiences (Belsher et al., 2012; Lepore, 2001). They also provide patients opportunities to openly communicate their physical and emotional unmet needs and can consequently provide support that reduces said unmet needs (Haber et al., 2007; Luszczynska et al., 2013; Martino, et al., 2019). Indeed, higher perceived social support is robustly associated with lower depressive symptoms (Carpenter, Fowler, Mawell et al., 2010; Fong et al., 2017; Gonzalez‐Saenz de Tejada et al., 2017; Hsieh et al., 2020; Hu et al., 2018; Karnell et al., 2007; Mehnert et al., 2010; Thompson, Pérez, Kreueter, Margenthaler et al., 2017) and greater life satisfaction (Dunn et al., 2013; Faraci & Bottaro, 2022; Hamdan-Mansour et al., 2015; Li, Lyu, Wang, Yin, Zhang, & Li, 2024; Stephens et al., 2010) among patients with various types of cancer.

On the other hand, patients may perceive social conditions that lead them to modify or restrict their disclosure of cancer-related thoughts, feelings, or concerns—a social context known as social constraint (Lepore, 2001; Wong et al., 2018). Social constraint intentionally or unintentionally reduces or disrupts opportunities for patients to emotionally and cognitively process their cancer-related experiences (Lepore, 2001). For example, patients may perceive that their social partner is avoiding discussion of cancer-related thoughts, minimizing or dismissing the patient’s cancer-related feelings, hiding their own cancer-related thoughts in an effort to protect the patient, or not seeming to understand the patient’s situation (Lepore & Ituarte, 1999). Among patients with various types of cancer, higher social constraint has been associated with greater depressive symptoms (Adams et al., 2015; Chambers et al., 2015; Cordova et al., 2001; Darabos & Hoyt, 2017; Hyland et al., 2019; Rivera Rivera & Burris, 2020), lower overall psychological well-being (Cordova et al., 2001; Manne et al., 1997; Pasipanodya et al., 2012; Rivera Rivera & Burris, 2020), and lower health-related quality of life (Cui, Wang, & Wang, 2021).

While theoretical distinctions between social support and social constraint have not been fully delineated, social support and social constraint have been conceptualized as positively and negatively valenced components of social functioning that may independently covary and co-occur in the same interaction or relationship (Rivera Rivera & Burris, 2020; Rivera Rivera, Badour, & Burris, 2021). For example, a patient may perceive high levels of both social support and social constraint in instances when their social partner is focused on “staying positive”—they might provide significant tangible support while simultaneously insisting that the patient not worry or think about their cancer. When considering how these two constructs might then be associated with constructs such as depressive symptoms and life satisfaction, psychometric models posit that constructs with similar underlying valence will be more strongly associated with each other than will constructs with different underlying valences (Chida & Steptoe, 2008; Cordova et al., 2007; Ingersoll-Dayton et al., 1997). This suggests that positive aspects of one’s social environment (e.g., social support) would be most strongly associated with positively valenced outcomes (e.g., life satisfaction), while negative aspects (e.g., social constraint) would correlate most strongly with negatively valenced outcomes (e.g., depressive symptoms). Empirical studies simultaneously examining both social support and social constraint in the same model (and thus controlling for each other’s effects) have indeed found that social support is a stronger predictor of indicators of positive well-being and adjustment, such as benefit finding (Dunn et al., 2011) and post-traumatic growth (Nenova et al., 2013). In contrast, social constraint is consistently the strongest or only predictor of indicators of maladaptive adjustment, such as post-traumatic stress (Swartzman et al., 2017; Widows et al., 2000), overall negative mental health symptoms (Shim et al., 2006; Wingard et al., 2010), role limitations due to emotional problems (Figueiredo et al., 2004), distress (Mosher et al., 2012), negative affect (Boinon et al., 2012), and depressive symptoms (Boinon et al., 2012; Schmidt & Andrykowski, 2004).

In addition to valence, two additional factors may also impact differences in associations between social support and social constraint and psychological adjustment. First, previous studies examining both social support and social constraint in the same model have inconsistently specified the source of support and constraint. Studies have specified for patients to consider support from their social network at large (Boinon et al., 2012; Figueiredo et al., 2004; Schmidt & Andrykowski, 2004; Shim et al., 2006; Wingard et al., 2010), their family at large (Swartzman et al., 2017), either a spouse, friend, or relative (Dunn et al., 2011), their most important source of support (Widows et al., 2000), or their spouse or romantic partner (Mosher et al., 2012; Nenova et al., 2013). Evidence has demonstrated that examining support from undistinguished sources yields negligible or inconsistent results (Luszczynska et al., 2013), and additional evidence has indicated that romantic partners in particular have a significant impact on patients’ cancer experiences that may be unique from that of other individuals in patients’ support networks (Mosher et al., 2012; Nenova et al., 2013). For various reasons, married or partnered patients tend to have better cancer diagnosis-, treatment-, and mortality-related outcomes (Ibrahimi & Pinherio, 2017; Wang et al., 2011; Zhang, Yang, Qiu, & Zhou, 2022) compared with single or widowed patients and many patients cite their spouse as their most valuable source of support (Pfaendler et al., 2015; Ruiz-Rodríguez et al., 2022). Similarly, perceiving social constraint from significant others has been found to be particularly distressing (Manne & Glassman, 2000), particularly for male patients (Zakowski et al., 2003). This study therefore focused specifically on support and constraint from patients’ spouses or romantic partners given the high frequency of social contact couples have.

Second, the degree to which patients benefit or suffer from social interactions may also depend on their gender given gender norms surrounding support-seeking and emotional disclosure (Ettridge et al., 2018; Fish et al., 2015). For example, female patients with various types of cancer, compared to male patients, tend to perceive greater levels of social constraint and avoidance (Lyons et al., 2022; Pedersen et al., 2011) and lower levels of social support (Pedersen et al., 2011) from their partners. However, male patients with prostate cancer, compared to female patients with gynecological cancers, reported significantly greater distress and intrusive thoughts in the face of perceived social constraint from their spouse–despite reporting comparable levels of social constraint with female patients (Zakowski et al., 2003). Similarly, in a nationally representative sample, men exhibited significant longitudinal declines in health-related quality of life in response to longitudinal declines in social support from their support network at large, whereas this was not the case for women (Hajek et al., 2016). Limited evidence therefore supports a potential gender moderating role in associations between psychological adjustment and different types of social factors such that, in the face of social contexts characterized by lower levels of social support and/or higher levels of social constraint, male patients’ psychological adjustment may be more negatively impacted compared to female patients.

This current study compares the extent to which social support and social constraint are associated with indicators of psychological adjustment and examines the putative moderating role of gender in such associations. This study utilized a sample with a single cancer type that is considered to be “gender neutral” to control for cancer-specific variability that may be introduced when examining more than one type of cancer, particularly to avoid comparing effects across “gender-specific” such as breast, gynecological, and prostate cancer. It was hypothesized that: (1) after controlling for social constraint, higher levels of social support would be associated with higher levels of life satisfaction, (2) after controlling for social support, higher levels of social constraint would be associated with higher levels of depressive symptoms, and (3) lower levels of social support and higher levels of social constraint would be more strongly associated with depressive symptoms in male patients compared with female patients. Gender differences in associations between life satisfaction and social support/social constraint were exploratory given the limited literature in this area.

## Methods

### Participants

Participants were 124 patients who were newly diagnosed with colorectal cancer. Eligibility criteria for patients included: (a) 18 years or older, (b) Diagnosed with stage I to IV colon or rectal cancer within 10 months prior to the enrollment, (c) Be able to read and speak English or Spanish at the 5th grade level, and (d) Have a romantic partner or spouse (hereafter, spouse) involved in care and daily activities. Exclusion criteria included (a) Active but untreated substance dependence, psychosis, dementia, sleeping disorders, phobias pertaining to medical treatment procedures, or suicidal ideation, (b) Unable to see or hear, (c) Poor physical functioning (defined as Eastern Cooperative Oncology Group scores ≥ 3 or Karnofsky Performance Status scores < 50; Oken et al., 1982; Karnofsky, 1949), (d) Poor cognitive functioning (Mini Mental State Examination < 24; Folstein et al., 1983), or (e) Under end-of-life care (< 6 months life expectancy). Patients were primarily middle-aged, heterosexual males who had an annual household income of more than $40,000 and more than a college degree. Patients were primarily Hispanic, which broadly represented the ethnic makeup of the recruiting area. Patients were enrolled in the study approximately 6 months post-diagnosis and mainly diagnosed at advanced stages. One patient identified as homosexual in the sample.

### Procedures

The current study is a secondary analysis of the first timepoint of a larger longitudinal study on the quality of life of patients with colorectal cancer and their spouse or romantic partner caregivers (R01NR016838). The study was conducted in compliance with the Institutional Review Boards of University of Miami, Sylvester Comprehensive Cancer Center Protocol Review Committee, and the Jackson Health System Institutional Review Board. Potentially-eligible patients were identified by medical records from participating oncology clinics. Patients were recruited in person or over the phone from ten clinics operated by Sylvester Comprehensive Cancer Center. Of the available pool of eligible patients, a total of 158 patient-caregiver dyads (57.66%) enrolled in the parent study that required both patients and their spouses/partners to be enrolled. Of those, 9 patients did not provide any data. An additional 25 patients were not given the measure of social constraint, which was added later, leaving a total of 124 patients in the current analysis. Eligible patients provided written informed consent prior to collecting study data. Patients completed self-report questionnaires, which included demographic questions and measures of perceived social support, perceived social constraint, depressive symptoms, and life satisfaction. Patients received $100 upon completion of all study measures.

### Measures

#### Perceived social support

The extent to which patients perceived various supportive behaviors from their spouse over the past month was measured by the 6-item positive support subscale of the Sources of Social Support Scale (SSSS; Carver, 2006) on a 5-point scale (1 = Not at all, 5 = A lot). Sample items include “How much does he/she give you reassurance, encouragement, and emotional support (affection), concerning your cancer?”, “How much does he/she give you advice or information about your cancer?”, and “How much does he/she give you assistance with things related to your cancer?”. Responses to individual items were averaged, with higher scores indicating greater perceived social support from their spouse. The six items had good internal consistency in the present study (α = 0.90).

#### Perceived social constraint

The extent to which patients perceived their social interactions with their spouse or partner to be unsupportive, dismissive, critical, or marked by misunderstanding over the past month was measured using the 15-item Social Constraints scale (Lepore & Ituarte, 1999) on a 4-point scale (1 = Never, 4 = Often). Sample items include “Did your spouse/partner act uncomfortable when you talked about your illness?,” “Did your spouse/partner tell you not to worry so much about your health?,” and “Did your spouse/partner seem to be hiding his/her feelings?”. Items were averaged, with higher scores indicating greater perceived social constraint from their spouse or partner. The 15 items had good internal consistency in the present study (α = 0.85).

#### Depressive symptoms

The degree to which patients experienced various depressive symptoms over the past month was measured by the 20-item Center for Epidemiologic Studies-Depression Scale (CES-D; Radloff, 1977) on a 4-point scale (0 = Rarely or none of the time; 3 = Most or all of the time). Items were summed after reversal coding when necessary to indicate higher scores for greater levels of depressive symptomology (Radloff, 1977). The 20 items had good internal consistency in the present study (α = 0.88).

#### Life satisfaction

The extent to which patients were satisfied with their lives was measured by the 5-item Satisfaction with Life Scale (SWLS; Diener et al., 1985), on a 7-point Likert scale (1 = Strongly Disagree, 7 = Strongly Agree). Items were summed with higher scores indicating greater satisfaction with life. The 5 items had good internal consistency in the present study (α = 0.88).

#### Covariates

Covariates known to be significantly associated with depressive symptoms and life satisfaction of patients with cancer, including age (Fagundes et al., 2014; Gana et al., 2013; Hinz et al., 2019; McAdams et al., 2012, Wen et al., 2019), income (Budría & Ferrer-I-Carbonell, 2019; Proto & Rustichini, 2015; Walker et al., 2021), and advanced cancer stage (Dunn et al., 2013; Hinz et al., 2019; Wen et al., 2019) were included in the analyses as covariates.

### Statistical analysis

Means, standard deviations, and frequencies of study variables were computed and reported in Table 1. Zero-order correlations among study variables were computed and reported in Table 2. Pearson’s product moment correlation coefficients were reported for continuous and normally distributed variables. Spearman’s rank correlation coefficients were reported for dichotomous and non-normally distributed variables. Hierarchical general linear modeling (GLM) was employed to examine the associations between social support and social constraint with psychological adjustment (i.e., depressive symptoms and life satisfaction) simultaneously. The covariates (i.e., age, income, and cancer stage) were included in the model first. Main study variables (i.e., social support, social constraint, and gender) were added to the model in the second step, followed by the two-way interaction terms between either social support or social constraint and gender in the third step. If interaction terms were significant, post-hoc tests were conducted to depict simple effects. Exploratory analyses examining the interaction effect between social support and social constraint, suggested by an anonymous reviewer, were also examined. All data analyses were conducted using SPSS Version 28.0 (IBM Corp., 2022).

**Table 1:** Sample Characteristics and Study Variables Descriptives

Variable	Total sample N = 124		Male patients N = 82		Female patients N = 42		*t* (122) or $${\chi }^{2}$$	*p*
	Mean or Frequency	SD or % of total sample	Mean or Frequency	SD or % of total sample	Mean or Frequency	SD or % of total sample		
Age (years)	56.62	10.66	57.58	10.44	54.74	10.96	1.409	0.161
Ethnicity								
Hispanic	78	62.9%	55	44.4%	23	18.5%	$${\chi }^{2}$$(4,124) = 6.308	0.177
Non-Hispanic White	33	25.8%	20	16.1%	13	10.5%		
Non-Hispanic Black	7	6.5%	5	4.0%	2	1.6%		
Other	6	4.8%	2	1.6%	4	3.2%		
Income								
< $10,000	11	8.9%	8	6.5%	3	2.4%	$${\chi }^{2}$$(5,124) = 1.033	0.960
$10,000–$39,999	21	16.8%	14	11.3%	7	5.6%		
$40,000–$69,999	25	20.2%	16	12.9%	9	7.3%		
$70,000–$120,000	25	20.1%	18	14.5%	7	5.6%		
> $120,000	29	23.4%	18	14.5%	11	8.9%		
Prefer not to answer	13	10.5%	8	6.5%	5	4.0%		
Education								
Less than High School	15	12.1%	12	9.7%	3	2.4%	$${\chi }^{2}$$(6,124) = 9.416	0.152
High School Diploma/GED	10	8.1%	8	6.5%	2	1.6%		
Associates/Technical Degree	18	14.5%	7	5.6%	11	8.9%		
Some College	13	10.5%	10	8.1%	3	2.4%		
College Degree	31	25.0%	21	16.9%	10	8.1%		
Professional/Graduate Degree	36	29.0%	23	18.5%	13	10.5%		
Did not answer	1	0.8%	1	0.8%	0	0%		
Cancer stage								
Stage I	18	14.5%	12	9.7%	6	4.8%	$${\chi }^{2}$$(3,124) = 6.179	0.103
Stage II	17	13.7%	9	7.3%	8	6.5%		
Stage III	51	41.1%	30	24.2%	21	16.9%		
Stage IV	38	30.6%	31	25.0%	7	5.6%		
Time since diagnosis (months)	6.72	3.91	6.67	3.88	6.83	4.00	− 0.221	0.826
Social support (1–5)	4.07	0.92	4.45	0.88	3.90	0.97	1.466	0.145
Social constraint (1–4)	1.50	0.45	1.44	0.40	1.61	0.52	− 2.054	0.042
Depressive symptoms (0–60)	13.41	9.13	12.30	8.70	15.60	9.66	− 1.919	0.057
Life satisfaction (5–35)	23.05	7.83	22.98	7.81	23.19	7.95	− 0.144	0.886

*N* = 124

**Table 2:** Zero-order correlation coefficients among study variables

	Age	Income	Advanced stage	Gender	Social support	Social constraint	Depressive symptoms
Income	− 0.297**	–					
Advanced stage	− 0.040	− 0.008	–				
Gender	− 0.127	0.033	− 0.217*	–			
Social support	− 0.060	0.075	0.081	− 0.132	–		
Social constraint	− 0.139	0.049	0.024	0.183*	− 0.397**	–	
Depressive symptoms	− 0.123	− 0.056	0.067	0.171	− 0.163	0.432**	–
Life satisfaction	0.022	0.096	− 0.181*	0.013	0.168	− 0.318**	− 0.594**

*N* = 124, * *p* < 0.05, ** p < 0.01

Pearson’s product moment correlation coefficients are reported for continuous and normally distributed variables. Spearman’s rank correlation coefficients are reported for dichotomous and nonnormally distributed variables. Income: ≥ $40,000 = 1; < $40,000 = 0; Advanced Stage: Cancer stages III and IV = 1; Cancer stages I and II = 0; Gender: Female = 1, Male = 0

## Results

### Sample characteristics

Table 1 displays sample characteristics for the total sample as well as separated by gender. On average, participants reported higher levels of social support than patients in another sample with breast cancer (*t*(252) = 11.96, *p* < 0.001: Kinsinger et al., 2011). Participants also reported levels of social constraint that were slightly lower than those of patients with breast cancer (*t*(167) = 1.85, *p* = 0.066: Pasipadonya et al., 2012) but higher than those of patients with prostate cancer (*t*(306) = 2.39, *p* = 0.018: Agustsdottir et al., 2010). Participants reported moderate levels of depressive symptoms that were comparable to a sample of patients with breast cancer (*t*(223) = 1.48, *p* = 0.14: Giese-Davis et al., 2011) but higher compared with a sample of patients with colorectal cancer (*t*(764) = 2.03, *p* = 0.04: Calman et al., 2021) and a sample of general older adults (*t*(1,664) = − 14.83, *p* < 0.0001: Andresen et al., 1994). Participants also reported lower life satisfaction compared to a sample of patients with primarily colorectal and breast cancer (*t*(835) = 6.89, *p* < 0.001: Lorenzo-Seva et al., 2019) and a nationally representative sample of adults (*t*(1,533) = 3.99, *p* < 0.001: Vazquez et al., 2015). Female participants reported significantly higher levels of perceived social constraint and marginally higher depressive symptoms, compared with male patients. Male and female participants in the current study reported comparable levels of social support and life satisfaction. Table 2 displays zero-order correlation coefficients among study variables.

### Associations of social support, social constraint, depressive symptoms, and life satisfaction

As shown in Table 3, hierarchical general linear modeling revealed that covariates were not significantly associated with depressive symptoms or life satisfaction. Among main study variables, only higher levels of social constraint were associated with higher levels of depressive symptoms and lower levels of life satisfaction. Further, a significant interaction between gender and social constraint on depressive symptoms indicated that this association differed by gender. Subsequent analyses indicated that higher levels of social constraint were associated with higher levels of depressive symptoms for male patients (*b* = 11.923, *p* < 0.001) but not for female patients (*b* = 3.860, *p* = 0.135). Finally, the interactions between social constraint and social support were insignificant when predicting depressive symptoms and life satisfaction (Table 3).

**Table 3:** Hierarchical general linear modeling predicting depressive symptoms and life satisfaction

	Depressive symptoms			Life satisfaction		
	$b (SE)$	*t*	*p*	$b (SE)$	*t*	*p*
**Covariates**	*R*^2^ = 0.035			*R*^2^ = 0.029		
Age	− 0.119 (0.081)	− 1.463	0.146	0.027 (0.070)	0.389	0.698
Income	2.466 (1.977)	1.247	0.215	− 2.388 (1.700)	− 1.405	0.163
Advanced cancer stage	− 2.144 (1.868)	− 1.148	0.253	2.316 (1.606)	1.442	0.152
**Main effects**	*ΔR*^*2*^ = 0.188			*ΔR*^*2*^ = 0.108		
Gender	− 1.974 (1.616)	− 1.221	0.224	− 1.027 (1.459)	− 0.704	0.483
Social support	0.118 (0.898)	0.132	0.895	0.433 (0.811)	0.535	0.594
Social constraint	8.320 (1.832)	4.541	< 0.001	− 5.399 (1.654)	− 3.264	0.001
**Two-way interaction effects**	*ΔR*^*2*^ = 0.038			*ΔR*^*2*^ = 0.015		
Gender * social support	0.443 (1.835)	0.242	0.809	1.210 (1.684)	0.718	0.474
Gender * social constraint	8.424 (3.637)	2.316	0.022	− 2.780 (3.338)	− 0.833	0.407
**Two-way interaction effect**	*ΔR*^*2*^ = 0.000			*ΔR*^*2*^ = 0.000		
Social support * social constraint	0.186 (1.810)	0.103	0.950	− 0.103 (1.634)	− 0.063	0.950

Income: ≥ $40,000 = 1; < $40,000 = 0; Advanced Cancer Stage: Cancer stages III and IV = 1; Cancer stages I and II = 0; Gender: Female = 1, Male = 0

## Discussion

The current study examined the extent to which social support and social constraint were associated with depressive symptoms and life satisfaction in patients who were recently diagnosed with colorectal cancer. Results revealed that greater social constraint was significantly associated with greater depressive symptoms, although further analyses revealed that this was the case only for male patients. Greater social constraint was also significantly associated with lower life satisfaction regardless of gender. Social support was not significantly associated with either outcome.

### Gender interactions: social constraint and depressive symptoms

In the full sample, social constraint was associated with depressive symptoms as expected; however, subsequent analyses by gender revealed that this association only held for male patients. The fact that social constraint was not significantly associated with female patients’ depressive symptoms was unexpected given that female patients in this sample reported significantly higher levels of social constraint compared with male patients, a gender difference that is in line with existing findings (Lyons et al., 2022; Pedersen et al., 2011). Such findings would need to be replicated before drawing firm conclusions from a null effect. However, there are several potential explanations for this pattern of findings given that patients were asked to evaluate perceived social support and social constraint specifically from their spouse and not from other individuals. First, in the face of coping with cancer, male patients are less likely than female patients to seek social support in general (Clarke et al., 2006; Henrich, 1999). Male patients are also more likely to have limited social networks and tend to rely primarily on their spouse for support (Clarke et al., 2006; Harrison et al., 1995; Pedersen et al., 2011; Salander & Hamberg, 2005), often forgoing individuals such as medical and mental health providers, family, and friends. Male patients’ limited social networks—when combined with feeling unable to disclose their thoughts and feelings to their partner—may place male patients at exacerbated risk for depressive symptoms (Fish et al., 2015; Park & Kim, 2021; Zakowski et al., 2003). In addition, male patients may be influenced by gender norms that discourage them from first having negative emotional reactions, followed by discouragement of displaying and disclosing such emotions to others (Boise & Hearn, 2017). Male patients may be more likely to perceive socially constraining behaviors that reinforce male gender norms and therefore feel discouraged to disclose negative emotional reactions to others (Carbone et al., 2024; Darabos & Hoyt, 2017; Ettridge et al., 2018; McCaughan et al., 2011), perhaps out of embarrassment, shame, desire to not appear weak, or need for control and independence (Ettridge et al., 2018; Fish et al., 2015). Especially considering the high proportion of Hispanic patients in this sample, it is also possible that gender-coded constructs culturally relevant to this population such as machismo and caballerismo (Badger et al., 2019; Nuñez et al., 2016; Yanez et al., 2016) are particularly salient. Machismo and caballerismo together encompass positive and negative traits, beliefs, and values characteristic of masculinity such as bravery, dominance, aggression, reserving one’s emotions, and emphasizing one’s role as the provider of the family (Nuñez et al., 2016). Such culturally-sensitive constructs may further reinforce male patients’ reluctance to disclose their thoughts and feelings. Overall, in order to target social constraint for male patients, it may be helpful to both increase male patients’ disclosure as well as decrease others’ behaviors that are perceived as constraining. Male patients may benefit from psychoeducation and other interventions that acknowledge cultural and societal challenges associated with self-disclosure, motivate patients to find individuals in their support network or support groups with whom they feel able to share their thoughts and emotions, teach specific communication skills that may facilitate such disclosure (Zhou et al., 2023b), and engage and provide similar psychoeducation to male patients’ support networks to encourage them to facilitate and accept such disclosure as well.

In contrast, female patients may feel freer to experience and share emotional reactions than male patients (Boise & Hearn, 2017). Female patients also tend to have larger social networks, meaning they have access to additional individuals with which to process their thoughts and feelings if they feel unable to do so with their spouse (Hann et al., 2002; Kroenke et al., 2006; Park & Kim, 2021). They can therefore still receive benefit from the cognitive and emotional processing posited to facilitate coping with cancer and alleviate depressive symptoms (Lepore, 2001), highlighting the importance of assessing levels of social support and social constraint as perceived from a multitude of individuals in patients’ networks.

### Critical role of social constraint for life satisfaction

Greater levels of social constraint were also significantly associated with lower levels of life satisfaction for both male and female patients. Although there has been limited research investigating the direct associations between social constraint and life satisfaction in cancer patients, findings are consistent with existing literature from the general population that evaluations of one’s social relationships are closely associated with evaluations of one’s life overall (Amati et al., 2018; Barger et al., 2009; Powdthavee, 2008). Having social relationships characterized by social constraint during a time of elevated stress, uncertainty, and need for support (Harrison et al., 2009; Sanson-Fisher et al., 2000) may be particularly salient when cancer patients evaluate their satisfaction with their life overall. In addition, findings should be interpreted with the caveat that this study specifically examined social constraint perceived from patients’ spouses given the indirect links between social constraint, romantic relationship functioning, and life satisfaction. For example, self-disclosure, a key component of social constraint, is also a key ingredient for maintaining relationship intimacy and closeness (Laurenceau et al., 1998; Sprecher & Hendrick, 2004), especially in couples coping with a shared stressor such as cancer (Manne et al., 2004; Zhou et al., 2023b). Lower marital satisfaction, relationship wellbeing, and marital adjustment in patients with cancer have robustly been associated with social constraint and self-disclosure (Manne et al., 2014; Pasipanodya et al., 2012; Porter et al., 2005; Soriano et al., 2021; Zhou et al., 2023b), as well as life satisfaction (Be et al., 2013; Buhler, Krauss, & Orth, 2021; Heller et al., 2004). This suggests that processes that disrupt or weaken a couple’s relationship (such as social constraint) may directly and indirectly lead to changes in life satisfaction. Findings support the growing development of communication-based couples-oriented interventions specifically targeting aspects of couples’ communication and romantic relationship such as reciprocal self-disclosure, partner responsiveness, relationship engagement, and relationship-compromising behaviors (Badr, 2017; Zhou et al., 2023a). Such interventions have already previously demonstrated positive impacts on individual-level functioning and quality of life for patients and their spouses in addition to positive impacts on relationship functioning (Zhou et al., 2023a) and in conjunction with the current study’s findings, warrant further development and wide-spread implementation to best support patients and their spouses.

### Social support and psychological adjustment

Lastly, social support was not significantly associated with depressive symptoms, consistent with the valence-specific hypothesis. However, contrary to our hypothesis, social support was also not found to be associated with life satisfaction. Although findings must be replicated to draw firmer conclusions, this finding is notable given social support’s seemingly robust protective role in supporting patient adjustment. Findings suggest that negative social interactions such as those characterized by social constraint may be more strongly associated with aspects of patients’ psychological adjustment regardless of valence. This pattern of findings is in line with Baumeister et al. (2001) observation that “bad is stronger than good” and suggests that greater focus be paid in psycho-oncology settings to assessing not just the presence or absence of social support, but also whether patients feel they have individuals in their support network with whom they can disclose and fully process their cancer-related feelings. However, there are several reasons to suspect that other types or aspects of social support may continue to play a role in patients’ depressive symptoms and life satisfaction.

First, perceptions of received social support (assessed in this study) and perceived availability of social support are distinct and only mildly correlated methods of measuring social support (Haber et al., 2007; Helgeson, 1993; Melrose et al., 2015). Indeed, perceived availability of support has been more consistently associated with psychological adjustment than received support in healthy samples (Helgeson, 1993; Melrose et al., 2015; Uchino, 2009), although evidence for this phenomenon has been more mixed in cancer populations (Luszczynska et al., 2013; Thompson et al., 2017; Schroevers et al., 2010). Further, given the high heterogeneity in available measures of social support (Rivera Rivera & Burris, 2020), this distinction between perceived availability and perceived receipt of support is not always fully delineated, further muddling existing findings.

Second, increasing attention has been paid towards whether a patient’s need for support is matched with the support they actually receive, a mismatch of which has consistently been associated with poor outcomes (Drageset et al, 2012; Linden & Vodermaier, 2012; Maisel & Gable, 2009; Merluzzi et al., 2016; Rini et al., 2011; Schroevers et al., 2010). For example, a patient with unmet support needs might desire support but not be receiving it, while another patient may have low support needs but receive an extreme level of support or receive the wrong type of support, which may threaten their sense of independence and self-efficacy (Ruiz-Rodriguez et al., 2022). It is also possible that patients are reporting lower levels of received social support due to having a low need for support rather than because they have support needs that are not being met. Consideration of patients’ nuanced support needs are therefore essential to consider when measuring social support, although there is a dearth of available measures assessing such support needs (Krumholz et al., 1998; Linden & Vodermaier, 2012).

Third, research has indicated that invisible social support, or support which is not perceived by the patient (Bolger et al., 2000)—and thus not accounted for by patients in their reports of received social support in the present study—may be more essential in dealing with a stressor that is outside of a patient’s control. Receiving invisible support has been found to be positively associated with patient adjustment (Bolger & Amarel, 2007; Bolger et al., 2000; Maisel & Gable, 2009; Nurullah, 2012), proposed in part because being aware of the support they are receiving may take an emotional toll on some patients (Bolger et al., 2000; Howland & Simpson, 2010). Overall, the large variability in conceptualizations and corresponding measures of social support may be contributing to inconsistencies in the literature regarding social support’s protective role in patient adjustment (Barrera, 1986; Rivera Rivera & Burris, 2020).

Fourth, cultural views and practices around social support and emotional disclosure have also been shown to differentiate findings (Nurullah, 2012; Wellisch, Kagawa-Singer, Reid, Lin, Nishikawa-Lee, & Wellisch, 1999), a consideration which may again be particularly salient given the high percentage of Hispanic patients in our sample (Gonzalez et al., 2005; Tyson et al., 2017). In addition to previously mentioned gender-coded constructs such as machismo and caballerismo, constructs relevant to Hispanic populations such as familism, or the cultural value that emphasizes supporting one’s family (Pedreira et al., 2024; Yanezet al., 2016) and acculturation status (Perez & Cruess, 2011; Stephens et al., 2010) may be especially relevant and have demonstrated mixed findings in regards to being helpful or harmful for mental health, such as when an individual may simultaneously feel guilty for receiving support from their family while also being more likely to perceive a higher availability of support (Perez & Cruess, 2011). More research is needed to understand how social support and social constraint may be differentially associated with depressive symptoms and life satisfaction based on culturally sensitive constructs.

Finally, it may be that social support from other individuals in their support network (other than their romantic partner) is more essential, or at least needs to be accounted for. It is possible that, although social constraint from patients’ spouses is overshadowing the possible salutary properties of social support from their spouses, receiving social support from other individuals in their support network may be able to overcome the negative associations of spousal social constraint. Overall, a number of factors may help explain the lack of significant associations with social support. Findings highlight the need for more comprehensive, multidimensional, and commonly utilized measures of social support (Rivera Rivera & Burris, 2020). Relevant dimensions of interest may include capturing support perceived from multiple sources from the patient’s social network, distinguishing perceived availability vs. perceptions of received support, and patient’s needs for support vs. the support they are actually receiving. Such information may aid in adding further nuance to the role social support plays in patient adjustment. In addition, despite the field’s strong focus on social support in past decades, findings support greater investigation of social constraint’s role in impacting patients’ individual-level and relationship-level functioning over and above what is able to be explained by social support.

### Limitations and future directions

Findings should be interpreted in light of study limitations. First, this study was cross-sectional, precluding conclusions on the directionality and causality between study variables. For example, common experiences of individuals experiencing depressive symptoms include the tendency to have negative cognitions which are thought to be involved in both the development and maintenance of depression (Teasdale, 1983), being rigid in their beliefs and focusing less on evidence that contradicts said negative cognitions (Aguilera et al., 2019; Lau, et al., 2020), and experiencing less positive affect overall (Sharpley et al., 2013). Therefore, if a patient was already experiencing depressive symptoms prior to their diagnosis or is experiencing depressive symptoms as a result of their diagnosis or other cancer-related experiences, they may also be more likely to perceive their spouse as socially constraining despite objective evidence indicating otherwise. Depressive symptoms can also lead to decreased engagement in behaviors that the patient previously found helpful and enjoyable, such as support seeking and disclosing their cancer-thoughts and feelings to their spouse, thereby exacerbating a tendency to perceive less support and greater social constraint from their spouse.

It could also be the case that findings change over time as the couple progresses through the cancer journey. Patients in this study were observed approximately six months following their diagnosis. Social support from family, friends, and health-care providers typically declines after a cancer patient’s initial diagnosis (Arora et al., 2007; Salonen et al., 2013). However, limited evidence suggests that spousal social constraint also typically declines over time (Soriano et al., 2017) as couples become more adjusted to the cancer experience. Studies examining longitudinal trajectories of social support and social constraint over the course of the patient’s cancer experience are essential to understanding directionality concerns and developing a better understanding of patients’ needs and concerns as they transition into survivorship.

Third, the sample size and smaller representation of female patients compared to male patients in this study limited the examination of additional factors associated with markers of patient adjustment and corresponding gender differences. The sample was also restricted to a specific cancer type (i.e., colon and rectal cancers), which may limit generalizability to other cancer types. However, choosing a gender-neutral cancer such as colon and rectal cancers allows for a more controlled examination of gender differences compared to confounding factors that are introduced from examining more gender-specific cancers such as breast and prostate cancer. Our sample was also primarily Hispanic, primarily heterosexual, and typically of higher economic status and educational background, which may limit generalizability to patients from different social, ethnic, economic, and sexual backgrounds and identities. Lastly, given that participation in the study required patients to have a romantic spouse or partner who served as their primary caregiver and who was also willing to participate in the study with them, generalizability to patients who do not have a romantic spouse or partner and patients who might be in lower quality romantic relationships is limited.

## Conclusion

This study provides additional evidence regarding the role that different aspects of patients’ social functioning have in the association with patient psychological adjustment, particularly negative social functioning characterized by social constraint. Findings also contribute further evidence regarding the importance gender may play in such associations and particularly, the importance of social functioning for male patients with cancer. Lastly, findings highlight the need for nuanced measurement of social functioning, different aspects of which may lead to different findings. Future studies and interventions may consider expanding to additional individuals in patients’ support networks, using multi-faceted measures of social functioning, and examining additional moderators of the associations between social and psychological functioning such as cultural, cognitive, emotional, biological, and disease-related factors.

## Data Availability

This study and its analysis plan was preregistered with an analysis plan (10.17605/OSF.IO/JU9DK). Deidentified data and analytic code used to conduct this study are not publicly available but can be provided upon request. Materials used to conduct this study are not publicly available.

## References

[CR1] Adams, R. N., Winger, J. G., & Mosher, C. E. (2015). A meta-analysis of the relationship between social constraints and distress in cancer patients. *Journal of Behavioral Medicine,**38*, 294–305. 10.1007/s10865-014-9601-6 25262383 10.1007/s10865-014-9601-6 PMC4355094

[CR2] Aguilera, M., Paz, C., Compañ, V., Medina, J. C., & Feixas, G. (2019). Cognitive rigidity in patients with depression and fibromyalgia. *International Journal of Clinical and Health Psychology,**19*, 160–164. 10.1016/j.ijchp.2019.02.002 31193143 10.1016/j.ijchp.2019.02.002 PMC6517680

[CR3] Agustsdottir, S., Kristinsdottir, A., Jonsdottir, K., Larusdottir, S. O., Smari, J., & Valdimarsdottir, H. B. (2010). The impact of dispositional emotional expressivity and social constraints on distress among prostate cancer patients in Iceland. *British Journal of Health Psychology,**15*, 51–61. 10.1348/135910709x426148 19341530 10.1348/135910709X426148

[CR4] Amati, V., Meggiolaro, S., Rivellini, G., & Zaccarin, S. (2018). Social relations and life satisfaction: The role of friends. *Genus,**74*, 1–18. 10.1186/s41118-018-0032-z 29755134 10.1186/s41118-018-0032-z PMC5937874

[CR5] American Cancer Society. (2024). *Cancer facts and figures 2024*. American Cancer Society. Retrieved from https://www.cancer.org/content/dam/cancer-org/research/cancer-facts-and-statistics/annual-cancer-facts-and-figures/2024/2024-cancer-facts-and-figures-acs.pdf

[CR6] Andresen, E. M., Malmgren, J. A., Carter, W. B., & Patrick, D. L. (1994). Screening for depression in well older adults: Evaluation of. *American Journal of Preventative Medicine,**10*, 77–84. 10.1016/s0749-3797(18)30622-6 8037935

[CR7] Arora, N. K., Finney Rutten, L. J., Gustafson, D. H., Moser, R., & Hawkins, R. P. (2007). Perceived helpfulness and impact of social support provided by family, friends, and health care providers to women newly diagnosed with breast cancer. *Psycho-Oncology,**16*, 474–486. 10.1002/pon.1084 16986172 10.1002/pon.1084

[CR8] Badr, H. (2017). New frontiers in couple-based interventions in cancer care: Refining the prescription for spousal communication. *Acta Oncologica,**56*, 139–145. 10.1080/0284186X.2016.1266079 27937437 10.1080/0284186X.2016.1266079

[CR150] Badger, T. A., Sikorskii, A., & Segrin, C. (2019) Contextual and cultural influences on caregivers of hispanic cancer suvivors. *Seminars in Oncology Nursing,**35*(4), 359–362. 10.1016/j.soncn.2019.06.008 10.1016/j.soncn.2019.06.008 31229343

[CR210] Baumeister, R. F., Bratslavsky, E., Finkenauer, C., & Vohs, K. D. (2001). Bad is stronger than good. *Review of General Psychology,**5*(4), 323–370. 10.1037/1089-2680.5.4.323

[CR9] Barger, S. D., Donoho, C. J., & Wayment, H. A. (2009). The relative contributions of race/ethnicity, socioeconomic status, health, and social relationships to life satisfaction in the United States. *Quality of Life Research,**18*, 179–189. 10.1007/s11136-008-9426-2 19082871 10.1007/s11136-008-9426-2 PMC6036920

[CR10] Barrera, I., & Spiegel, D. (2014). Review of psychotherapeutic interventions on depression in cancer patients and their impact on disease progression. *International Review of Psychiatry,**26*, 31–43. 10.3109/09540261.2013.864259 24716499 10.3109/09540261.2013.864259

[CR11] Barrera, M., Jr. (1986). Distinctions between social support concepts, measures, and models. *American Journal of Community Psychology,**14*, 413–445. 10.1007/bf00922627

[CR12] Be, D., Whisman, M. A., & Uebelacker, L. A. (2013). Prospective associations between marital adjustment and life satisfaction. *Personal Relationships,**20*, 728–739. 10.1111/pere.12011

[CR13] Belsher, B. E., Ruzek, J. I., Bongar, B., & Cordova, M. J. (2012). Social constraints, posttraumatic cognitions, and posttraumatic stress disorder in treatment-seeking trauma survivors: Evidence for a social-cognitive processing model. *Psychological Trauma: Theory, Research, Practice, and Policy,**4*, 386. 10.1037/a0024362

[CR14] Boinon, D., Sultan, S., Charles, C., Rosberger, Z., Delaloge, S., & Dauchy, S. (2012). How social sharing and social support explain distress in breast cancer after surgery: The role of alexithymia. *Journal of Psychosocial Oncology,**30*, 573–592. 10.1080/07347332.2012.703769 22963184 10.1080/07347332.2012.703769

[CR15] Bolger, N., & Amarel, D. (2007). Effects of social support visibility on adjustment to stress: Experimental evidence. *Journal of Personality and Social Psychology,**92*, 458. 10.1037/0022-3514.92.3.458 17352603 10.1037/0022-3514.92.3.458

[CR16] Bolger, N., Zuckerman, A., & Kessler, R. C. (2000). Invisible support and adjustment to stress. *Journal of Personality and Social Psychology,**79*, 953. 10.1037/0022-3514.79.6.953 11138764 10.1037//0022-3514.79.6.953

[CR17] Budría, S., & Ferrer-I-Carbonell, A. (2019). Life satisfaction, income comparisons, and individual traits. *Review of Income and Wealth,**65*, 337–357. 10.1111/roiw.12353

[CR18] Bühler, J. L., Krauss, S., & Orth, U. (2021). Development of relationship satisfaction across the life span: A systematic review and meta-analysis. *Psychological Bulletin,**147*, 1012. 10.1037/bul0000342 34928690 10.1037/bul0000342

[CR19] Büssing, A., Fischer, J., Haller, A., Heusser, P., Ostermann, T., & Matthiessen, P. F. (2009). Validation of the brief multidimensional life satisfaction scale in patients with chronic diseases. *European Journal of Medical Research,**14*, 171–177. 10.1186/2047-783x-14-4-171 19380290 10.1186/2047-783X-14-4-171 PMC3401007

[CR20] Calman, L., Turner, J., Fenlon, D., Permyakova, N. V., Wheelwright, S., Patel, M., Din, A., Winter, J., Richardson, A., Smith, P. W., & Foster, C. (2021). Prevalence and determinants of depression up to 5 years after colorectal cancer surgery: Results from the ColoREctal Wellbeing (CREW) study. *Colorectal Disease,**23*, 3234–3250. 10.1111/codi.15949 34679253 10.1111/codi.15949 PMC9298990

[CR22] Carbone, E., Loewenstein, G., Scopelliti, I., & Vosgerau, J. (2024). He said, she said: Gender differences in the disclosure of positive and negative information. *Journal of Experimental Social Psychology,**110*, 104525. 10.1016/j.jesp.2023.104525

[CR23] Carpenter, K. M., Fowler, J. M., Maxwell, G. L., & Andersen, B. L. (2010). Direct and buffering effects of social support among gynecologic cancer survivors. *Annals of Behavioral Medicine,**39*, 79–90. 10.1007/s12160-010-9160-1 20151235 10.1007/s12160-010-9160-1 PMC3645934

[CR24] Caruso, R., Nanni, M. G., Riba, M., Sabato, S., Mitchell, A. J., Croce, E., & Grassi, L. (2017). Depressive spectrum disorders in cancer: Prevalence, risk factors, and screening for depression: A critical review. *Acta Oncologica,**56*, 146–155. 10.1007/s12160-010-9160-1 28140731 10.1080/0284186X.2016.1266090

[CR25] Carver, C. S. (2006). Sources of social support scale. Retrieved from http://www.psy.miami.edu/faculty/ccarver/sclSSSS.phtml

[CR26] Chambers, S. K., Baade, P., Youl, P., Aitken, J., Occhipinti, S., Vinod, S., Valery, P. C., Garvey, G., Fong, K. M., Ball, D., & Zorbas, H. (2015). Psychological distress and quality of life in lung cancer: The role of health-related stigma, illness appraisals, and social constraints. *Psycho-Oncology,**24*, 1569–1577. 10.1002/pon.3829 25920906 10.1002/pon.3829 PMC5029590

[CR27] Chida, Y., & Steptoe, A. (2008). Positive psychological well-being and mortality: A quantitative review of prospective observational studies. *Psychosomatic Medicine,**70*, 741–756. 10.1097/psy.0b013e31818105ba 18725425 10.1097/PSY.0b013e31818105ba

[CR28] Chochinov, H. M. (2001). Depression in cancer patients. *The Lancet Oncology,**2*, 499–505. 10.1016/s1470-2045(01)00456-9 11905726 10.1016/S1470-2045(01)00456-9

[CR29] Clarke, S. A., Booth, L., Velikova, G., & Hewison, J. (2006). Social support: Gender differences in cancer patients in the United Kingdom. *Cancer Nursing,**29*, 66–72. 10.1097/00002820-200601000-00012 16557124 10.1097/00002820-200601000-00012

[CR30] Cordova, M. J., Cunningham, L. L., Carlson, C. R., & Andrykowski, M. A. (2001). Social constraints, cognitive processing, and adjustment to breast cancer. *Journal of Consulting and Clinical Psychology,**69*, 706. 10.1037/0022-006X.69.4.706 11550737

[CR31] Cordova, M. J., Giese-Davis, J., Golant, M., Kronenwetter, C., Chang, V., & Spiegel, D. (2007). Breast cancer as trauma: Posttraumatic stress and posttraumatic growth. *Journal of Clinical Psychology in Medical Settings,**14*, 308–319. 10.1007/s10880-007-9083-6

[CR32] Cui, C., Wang, L., & Wang, X. (2021). Health-related quality of life and social constraints among Chinese breast cancer patients: A cross-sectional study. *Health and Quality of Life Outcomes,**19*, 1–8. 10.1186/s12955-021-01871-0 34641883 10.1186/s12955-021-01871-0 PMC8507183

[CR33] Darabos, K., & Hoyt, M. A. (2017). Masculine norms about emotionality and social constraints in young and older adult men with cancer. *Journal of Behavioral Medicine,**40*, 259–270. 10.1007/s10865-016-9739-5 27033539 10.1007/s10865-016-9739-5 PMC5553069

[CR34] De Boise, S., & Hearn, J. (2017). Are men getting more emotional? Critical sociological perspectives on men, masculinities, and emotions. *The Sociological Review,**65*, 779–796. 10.1177/0038026116686500

[CR35] De Ridder, D., Geenen, R., Kuijer, R., & Van Middendorp, H. (2008). Psychological adjustment to chronic disease. *The Lancet,**372*, 246–255. 10.1016/s0140-6736(08)61078-8 10.1016/S0140-6736(08)61078-8 18640461

[CR36] Dezutter, J., Offenbaecher, M., Vallejo, M. A., Vanhooren, S., Thauvoye, E., & Toussaint, L. (2017). Chronic pain care: The importance of a biopsychosocial-existential approach. *The International Journal of Psychiatry in Medicine,**51*, 563–575. 10.1177/0091217417696738 10.1177/0091217417696738 28629291

[CR37] Diener, E. D., Emmons, R. A., Larsen, R. J., & Griffin, S. (1985). The satisfaction with life scale. *Journal of Personality Assessment,**49*, 71–75. 10.1207/s15327752jpa4901_13 16367493 10.1207/s15327752jpa4901_13

[CR38] Drageset, S., Lindstrøm, T. C., Giske, T., & Underlid, K. (2012). “The support I need”: Women’s experiences of social support after receiving a breast cancer diagnosis and awaiting surgery. *Cancer Nursing,**35*, E39–E47. 10.1097/ncc.0b013e31823634aa 22134160 10.1097/NCC.0b013e31823634aa

[CR39] Dunn, J., Ng, S. K., Breitbart, W., Aitken, J., Youl, P., Baade, P. D., & Chambers, S. K. (2013). Health-related quality of life and life satisfaction in colorectal cancer survivors: Trajectories of adjustment. *Health and Quality of Life Outcomes,**11*, 1–8. 10.1186/1477-7525-11-46 23497387 10.1186/1477-7525-11-46 PMC3648454

[CR40] Dunn, J., Occhipinti, S., Campbell, A., Ferguson, M., & Chambers, S. K. (2011). Benefit finding after cancer: The role of optimism, intrusive thinking, and social environment. *Journal of Health Psychology,**16*, 169–177. 10.1177/1359105310371555 20656765 10.1177/1359105310371555

[CR41] El Ibrahimi, S., & Pinheiro, P. S. (2017). The effect of marriage on stage at diagnosis and survival in women with cervical cancer. *Psycho-Oncology,**26*, 704–710. 10.1002/pon.4070 26810012 10.1002/pon.4070

[CR42] Ellis, E. M., Nelson, W. L., & Ferrer, R. A. (2019). Trajectories of current and predicted satisfaction with one’s life following a cancer diagnosis. *Annals of Behavioral Medicine,**53*, 158–168. 10.1093/abm/kay025 29746628 10.1093/abm/kay025 PMC6341020

[CR43] Ettridge, K. A., Bowden, J. A., Chambers, S. K., Smith, D. P., Murphy, M., Evans, S. M., Roder, D., & Miller, C. L. (2018). “Prostate cancer is far more hidden”: Perceptions of stigma, social isolation, and help-seeking among men with prostate cancer. *European Journal of Cancer Care,**27*, e12790. 10.1111/ecc.12790 29112317 10.1111/ecc.12790

[CR44] Fagundes, C., Jones, D., Vichaya, E., Lu, C., & Cleeland, C. S. (2014). Socioeconomic status is associated with depressive severity among patients with advanced non–small-cell lung cancer: Treatment setting and minority status do not make a difference. *Journal of Thoracic Oncology,**9*, 1459–1463. 10.1097/jto.0000000000000284 25170640 10.1097/JTO.0000000000000284 PMC4514021

[CR45] Faraci, P., & Bottaro, R. (2022). Association between perceived social support, illness perception, life orientation, life satisfaction, and quality of life within a sample of cancer patients. *International Journal of Psychological Research,**15*, 9–19. 10.21500/20112084.5263 36199528 10.21500/20112084.5263 PMC9529281

[CR46] Figueiredo, M. I., Fries, E., & Ingram, K. M. (2004). The role of disclosure patterns and unsupportive social interactions in the well-being of breast cancer patients. *Psycho-Oncology,**13*, 96–105. 10.1002/pon.717 14872528 10.1002/pon.717

[CR47] Fish, J. A., Prichard, I., Ettridge, K., Grunfeld, E. A., & Wilson, C. (2015). Psychosocial factors that influence men’s help-seeking for cancer symptoms: A systematic synthesis of mixed methods research. *Psycho-Oncology,**24*, 1222–1232. 10.1002/pon.3912 26202128 10.1002/pon.3912

[CR48] Folstein, M. F., Robins, L. N., & Helzer, J. E. (1983). The mini-mental state examination. *Archives of General Psychiatry,**40*, 812–812. 10.1001/archpsyc.1983.01790060110016 6860082 10.1001/archpsyc.1983.01790060110016

[CR49] Fong, A. J., Scarapicchia, T. M., McDonough, M. H., Wrosch, C., & Sabiston, C. M. (2017). Changes in social support predict emotional well-being in breast cancer survivors. *Psycho-Oncology,**26*, 664–671. 10.1002/pon.4064 26818101 10.1002/pon.4064

[CR50] Gana, K., Bailly, N., Saada, Y., Joulain, M., & Alaphilippe, D. (2013). Does life satisfaction change in old age: Results from an 8-year longitudinal study. *Journals of Gerontology,**68*, 540–552. 10.1093/geronb/gbs093 10.1093/geronb/gbs093 23103381

[CR51] Giese-Davis, J., Collie, K., Rancourt, K. M., Neri, E., Kraemer, H. C., & Spiegel, D. (2011). Decrease in depression symptoms is associated with longer survival in patients with metastatic breast cancer: A secondary analysis. *Journal of Clinical Oncology,**29*, 413–420. 10.1200/jco.2010.28.4455 21149651 10.1200/JCO.2010.28.4455 PMC3058287

[CR157] Gonzalez, G., Gallardo, N., & Bastani, R. (2005). A pilot study to define social support among Spanish-speaking women diagnosed with a breast abnormality suspicious for cancer: A brief research report. *Journal of Psychosocial Oncology,**23*(1), 109–120. 10.1300/J077v23n01_07 10.1300/J077v23n01_07 16492647

[CR52] González, P., Nuñez, A., Merz, E., Brintz, C., Weitzman, O., Navas, E. L., Camacho, A., Buelna, C., Penedo, F. J., Wassertheil-Smoller, S., & Perreira, K. (2017). Measurement properties of the Center for Epidemiologic Studies Depression Scale (CES-D 10): Findings from HCHS/SOL. *Psychological Assessment,**29*, 372. 10.1037/pas0000330 27295022 10.1037/pas0000330 PMC5154787

[CR53] Haber, M. G., Cohen, J. L., Lucas, T., & Baltes, B. B. (2007). The relationship between self-reported received and perceived social support: A meta-analytic review. *American Journal of Community Psychology,**39*, 133–144. 10.1007/s10464-007-9100-9 17308966 10.1007/s10464-007-9100-9

[CR54] Hajek, A., Brettschneider, C., Lange, C., Posselt, T., Wiese, B., Steinmann, S., Weyerer, S., Werle, J., Pentzek, M., Fuchs, A., & Stein, J. (2016). Gender differences in the effect of social support on health-related quality of life: Results of a population-based prospective cohort study in old age in Germany. *Quality of Life Research,**25*, 1159–1168. 10.1007/s11136-015-1166-5 26506992 10.1007/s11136-015-1166-5

[CR55] Hamdan-Mansour, A. M., Al Abeiat, D. D., Alzoghaibi, I. N., Ghannam, B. M., & Hanouneh, S. I. (2015). Psychosocial and sociodemographic correlates of life satisfaction among patients diagnosed with cancer in Jordan. *Journal of Cancer Education,**30*, 31–36. 10.1007/s13187-014-0678-y 24876063 10.1007/s13187-014-0678-y

[CR152] Hann, D., Baker, F., Denniston, M., Gesme, D., Reding, D., Flynn, T., ... & Kieltyka, R. L. (2002). The influence of social support on depressive symptoms in cancer patients: age and gender differences. *Journal of Psychosomatic Research,* **52**(5), 279–283. 10.1016/S0022-3999(01)00235-5 10.1016/s0022-3999(01)00235-5 12023124

[CR56] Harrison, J., Maguire, P., & Pitceathly, C. (1995). Confiding in crisis: Gender differences in pattern of confiding among cancer patients. *Social Science & Medicine,**41*, 1255–1260. 10.1016/0277-9536(94)00411-l 8545678 10.1016/0277-9536(94)00411-l

[CR57] Harrison, J. D., Young, J. M., Price, M. A., Butow, P. N., & Solomon, M. J. (2009). What are the unmet supportive care needs of people with cancer? A systematic review. *Supportive Care in Cancer,**17*, 1117–1128. 10.1007/s00520-009-0615-5 19319577 10.1007/s00520-009-0615-5

[CR58] Hartung, T. J., Brähler, E., Faller, H., Härter, M., Hinz, A., Johansen, C., Keller, M., Koch, U., Schulz, H., Weis, J., & Mehnert, A. (2017). The risk of being depressed is significantly higher in cancer patients than in the general population: Prevalence and severity of depressive symptoms across major cancer types. *European Journal of Cancer,**72*, 46–53. 10.1016/j.ejca.2016.11.017 28024266 10.1016/j.ejca.2016.11.017

[CR59] Helgeson, V. S. (1993). Two important distinctions in social support: Kind of support and perceived versus received. *Journal of Applied Social Psychology,**23*, 825–845. 10.1111/j.1559-1816.1993.tb01008.x

[CR60] Heller, D., Watson, D., & Ilies, R. (2004). The role of person versus situation in life satisfaction: A critical examination. *Psychological Bulletin,**130*, 574. 10.1037/0033-2909.130.4.574 15250814 10.1037/0033-2909.130.4.574

[CR211] Henrich, M. K. G. (1999). Illness-related distress: Does it mean the same for men and women?: Gender aspects in cancer patients’ distress and adjustment. *Acta Oncologica,**38*(6), 747–755. 10.1080/028418699432905 10.1080/028418699432905 10522765

[CR61] Hinz, A., Herzberg, P. Y., Lordick, F., Weis, J., Faller, H., Brähler, E., Härter, M., Wegscheider, K., Geue, K., & Mehnert, A. (2019). Age and gender differences in anxiety and depression in cancer patients compared with the general population. *European Journal of Cancer Care,**28*, e13129. 10.1111/ecc.13129 31290218 10.1111/ecc.13129

[CR62] Howland, M., & Simpson, J. A. (2010). Getting in under the radar: A dyadic view of invisible support. *Psychological Science,**21*, 1878–1885. 10.1177/0956797610388817 21097721 10.1177/0956797610388817

[CR63] Hsieh, Y. P., Roh, S., & Lee, Y. S. (2020). Spiritual well-being, social support, and depression among American Indian women cancer survivors: The mediating effect of perceived quality of life. *Families in Society,**101*, 83–94. 10.1177/1044389419853113

[CR64] Hu, T., Xiao, J., Peng, J., Kuang, X., & He, B. (2018). Relationship between resilience, social support, as well as anxiety/depression of lung cancer patients: A cross-sectional observation study. *Journal of Cancer Research and Therapeutics,**14*, 72–77. 10.4103/jcrt.jcrt_849_17 29516963 10.4103/jcrt.JCRT_849_17

[CR65] Hyland, K. A., Small, B. J., Gray, J. E., Chiappori, A., Creelan, B. C., Tanvetyanon, T., Nelson, A. M., Cessna-Palas, J., Jim, H. S., & Jacobsen, P. B. (2019). Loneliness as a mediator of the relationship of social cognitive variables with depressive symptoms and quality of life in lung cancer patients beginning treatment. *Psycho-Oncology,**28*, 1234–1242. 10.1002/pon.5072 30932275 10.1002/pon.5072

[CR148] IBM Corp. Released 2022. IBM SPSS Statistics for Windows, Version 26.0. Armonk, NY: IBM Corp.

[CR66] Ingersoll-Dayton, B., Morgan, D., & Antonucci, T. (1997). The effects of positive and negative social exchanges on aging adults. *The Journals of Gerontology,**52*, S190–S199. 10.1093/geronb/52b.4.s190 9224447 10.1093/geronb/52b.4.s190

[CR67] Karnell, L. H., Christensen, A. J., Rosenthal, E. L., Magnuson, J. S., & Funk, G. F. (2007). Influence of social support on health-related quality of life outcomes in head and neck cancer. *Head & Neck,**29*, 143–146. 10.1002/hed.20501 17111431 10.1002/hed.20501

[CR68] Karnofsky, D. A. (1949). The clinical evaluation of chemotherapeutic agents in cancer. *Evaluation of Chemotherapeutic Agents,**49*, 191–205. 10.3322/canjclin.18.2.72

[CR69] Kinsinger, S. W., Laurenceau, J. P., Carver, C. S., & Antoni, M. H. (2011). Perceived partner support and psychosexual adjustment to breast cancer. *Psychology & Health,**26*, 1571–1588. 10.1080/08870446.2010.533771 21598184 10.1080/08870446.2010.533771

[CR70] Krebber, A. M. H., Buffart, L. M., Kleijn, G., Riepma, I. C., De Bree, R., Leemans, C. R., Becker, A., Brug, J., Van Straten, A., Cuijpers, P., & Verdonck-de Leeuw, I. (2014). Prevalence of depression in cancer patients: A meta-analysis of diagnostic interviews and self-report instruments. *Psycho-Oncology,**23*, 121–130. 10.1002/pon.3409 24105788 10.1002/pon.3409 PMC4282549

[CR71] Kroenke, C. H., Kubzansky, L. D., Schernhammer, E. S., Holmes, M. D., & Kawachi, I. (2006). Social networks, social support, and survival after breast cancer diagnosis. *Journal of Clinical Oncology,**24*, 1105–1111. 10.1200/jco.2005.04.2846 16505430 10.1200/JCO.2005.04.2846

[CR72] Krumholz, H. M., Butler, J., Miller, J., Vaccarino, V., Williams, C. S., Mendes de Leon, C. F., Seeman, T. E., Kasl, S. V., & Berkman, L. F. (1998). Prognostic importance of emotional support for elderly patients hospitalized with heart failure. *Circulation,**97*, 958–964. 10.1161/01.CIR.97.10.958 9529263 10.1161/01.cir.97.10.958

[CR73] Lau, B. H., Chow, A. Y., Ng, T. K., Fung, Y. L., Lam, T. C., So, T. H., Chan, J. S., Chan, C. H., Zhou, J., Tam, M. Y., & Tsang, M. W. (2020). Comparing the efficacy of integrative body-mind-spirit intervention with cognitive behavioral therapy in patient-caregiver parallel groups for lung cancer patients using a randomized controlled trial. *Journal of Psychosocial Oncology,**38*, 389–405. 10.1080/07347332.2020.1722981 32146876 10.1080/07347332.2020.1722981

[CR212] Laurenceau, J. P., Barrett, L. F., & Pietromonaco, P. R. (1998). Intimacy as an interpersonal process: the importance of self-disclosure, partner disclosure, and perceived partner responsiveness in interpersonal exchanges. *Journal of Personality and Social Psychology,**74*(5), 1238. 10.1037/0022-3514.74.5.1238 10.1037//0022-3514.74.5.1238 9599440

[CR74] Lepore, S. J. (2001). A social–cognitive processing model of emotional adjustment to cancer. In A. Baum & B. L. Andersen (Eds.), *Psychosocial interventions for cancer.* (pp. 99–116). Washington: American Psychological Association. 10.1037/10402-006

[CR75] Lepore, S. J., & Ituarte, P. H. (1999). Optimism about cancer enhances mood by reducing negative social interactions. *Cancer Research, Therapy and Control,**8*, 165–174.

[CR76] Li, H., Lyu, M., Wang, A., Yin, Y., Zhang, J., & Li, P. (2024). Social support and life satisfaction in women with cervical cancer: A serial multiple mediation model. *Cancer Nursing,**47*(1), 64–71. 10.1097/ncc.0000000000001146 36322694 10.1097/NCC.0000000000001146

[CR77] Linden, W., & Vodermaier, A. (2012). Mismatch of desired versus perceived social support and associated levels of anxiety and depression in newly diagnosed cancer patients. *Supportive Care in Cancer,**20*, 1449–1456. 10.1007/s00520-011-1228-3 21744030 10.1007/s00520-011-1228-3

[CR78] Lorenzo-Seva, U., Calderon, C., Ferrando, P. J., del Mar Muñoz, M., Beato, C., Ghanem, I., Castelo, B., Carmona-Bayonas, A., Hernández, R., & Jiménez-Fonseca, P. (2019). Psychometric properties and factorial analysis of invariance of the Satisfaction with Life Scale (SWLS) in cancer patients. *Quality of Life Research,**28*, 1255–1264. 10.1007/s11136-019-02106-y 30644028 10.1007/s11136-019-02106-y

[CR79] Luszczynska, A., Pawlowska, I., Cieslak, R., Knoll, N., & Scholz, U. (2013). Social support and quality of life among lung cancer patients: A systematic review. *Psycho-Oncology,**22*, 2160–2168. 10.1002/pon.3218 23097417 10.1002/pon.3218

[CR147] Lyons, K. S., Gorman, J. R., Larkin, B. S., Duncan, G., & Hayes-Lattin, B. (2022). Active engagement, protective buffering, and depressive symptoms in Young-Midlife couples surviving cancer: The roles of age and sex. *Frontiers in Psychology,**13*, 816626. 10.3389/fpsyg.2022.816626 35250747 10.3389/fpsyg.2022.816626 PMC8891218

[CR80] Maisel, N. C., & Gable, S. L. (2009). The paradox of received social support: The importance of responsiveness. *Psychological Science,**20*, 928–932. 10.1111/j.1467-9280.2009.02388.x 19549083 10.1111/j.1467-9280.2009.02388.x

[CR81] Manne, S., & Glassman, M. (2000). Perceived control, coping efficacy, and avoidance coping as mediators between spousal unsupportive behaviors and psychological distress. *Health Psychology,**19*, 155. 10.1037/0278-6133.19.2.155 10762099 10.1037//0278-6133.19.2.155

[CR82] Manne, S., Kashy, D. A., Siegel, S., Myers Virtue, S., Heckman, C., & Ryan, D. (2014). Unsupportive partner behaviors, social-cognitive processing, and psychological outcomes in couples coping with early stage breast cancer. *Journal of Family Psychology,**28*, 214. 10.1037/a0036053 24611691 10.1037/a0036053 PMC4050663

[CR83] Manne, S., Sherman, M., Ross, S., Ostroff, J., Heyman, R. E., & Fox, K. (2004). Couples’ support-related communication, psychological distress, and relationship satisfaction among women with early stage breast cancer. *Journal of Consulting and Clinical Psychology,**72*, 660. 10.1037/0022-006x.72.4.660 15301651 10.1037/0022-006X.72.4.660

[CR84] Manne, S. L., Taylor, K. L., Dougherty, J., & Kemeny, N. (1997). Supportive and negative responses in the partner relationship: Their association with psychological adjustment among individuals with cancer. *Journal of Behavioral Medicine,**20*, 101–125. 10.1023/a:1025574626454 9144035 10.1023/a:1025574626454

[CR85] Manning, M., & Bettencourt, B. A. (2011). Depression and medication adherence among breast cancer survivors: Bridging the gap with the theory of planned behavior. *Psychology & Health,**26*, 1173–1187. 10.1080/08870446.2010.542815 21929477 10.1080/08870446.2010.542815

[CR86] Martino, M. L., Gargiulo, A., Lemmo, D., Dolce, P., Barberio, D., Abate, V., Avino, F., & Tortoriello, R. (2019). Longitudinal effect of emotional processing on psychological symptoms in women under 50 with breast cancer. *Health Psychology Open,**6*, 2055102919844501. 10.1177/2055102919844501 31037219 10.1177/2055102919844501 PMC6475855

[CR87] Massie, M. J. (2004). Prevalence of depression in patients with cancer. *JNCI Monographs,**2004*, 57–71. 10.1093/jncimonographs/lgh014 10.1093/jncimonographs/lgh014 15263042

[CR88] McAdams, K. K., Lucas, R. E., & Donnellan, M. B. (2012). The role of domain satisfaction in explaining the paradoxical association between life satisfaction and age. *Social Indicators Research,**109*, 295–303. 10.1007/s11205-011-9903-9

[CR151] McCaughan, E., Prue, G., Parahoo, K., McIlfatrick, S., & McKenna, H. (2011). Exploring and comparing the experience and coping behaviour of men and women with colorectal cancer at diagnosis and during surgery. *Journal of Advanced Nursing,**67*(7), 1591–1600. 10.1111/j.1365-2648.2010.05594.x 10.1111/j.1365-2648.2010.05594.x 21332577

[CR155] Merluzzi, T. V., Philip, E. J., Yang, M., & Heitzmann, C. A. (2016). Matching of received social support with need for support in adjusting to cancer and cancer survivorship. *Psycho‐Oncology,**25*(6), 684–690. 10.1002/pon.3896 10.1002/pon.3896 PMC4960824 26126444

[CR89] Mehnert, A., Lehmann, C., Graefen, M., Huland, H., & Koch, U. (2010). Depression, anxiety, post-traumatic stress disorder, and health-related quality of life and its association with social support in ambulatory prostate cancer patients. *European Journal of Cancer Care,**19*, 736–745. 10.1111/j.1365-2354.2009.01117.x 19832893 10.1111/j.1365-2354.2009.01117.x

[CR90] Melrose, K. L., Brown, G. D., & Wood, A. M. (2015). When is received social support related to perceived support and well-being? When it is needed. *Personality and Individual Differences,**77*, 97–105. 10.1016/j.paid.2014.12.047

[CR91] Mitchell, A. J., Chan, M., Bhatti, H., Halton, M., Grassi, L., Johansen, C., & Meader, N. (2011). Prevalence of depression, anxiety, and adjustment disorder in oncological, haematological, and palliative-care settings: A meta-analysis of 94 interview-based studies. *The Lancet Oncology,**12*(2), 160–174. 10.1016/s1470-2045(11)70002-x 21251875 10.1016/S1470-2045(11)70002-X

[CR92] Mosher, C. E., Lepore, S. J., Wu, L., Austin, J., Valdimarsdottir, H., Rowley, S., Isola, L., Redd, W. H., & Rini, C. (2012). Social correlates of distress following hematopoietic stem cell transplantation: Exploring the role of loneliness and cognitive processing. *Journal of Health Psychology,**17*, 1022–1032. 10.1177/1359105311432490 22253329 10.1177/1359105311432490 PMC3760721

[CR93] Nenova, M., DuHamel, K., Zemon, V., Rini, C., & Redd, W. H. (2013). Posttraumatic growth, social support, and social constraint in hematopoietic stem cell transplant survivors. *Psycho-Oncology,**22*, 195–202. 10.1002/pon.2073 21972000 10.1002/pon.2073 PMC3760719

[CR149] Nuñez, A., González, P., Talavera, G. A., Sanchez-Johnsen, L., Roesch, S. C., Davis, S. M., Arguelles, W., Womack, V. Y., Ostrovsky, N. W., Ojeda, L., Penedo, F., & Gallo, L. C. (2016). Machismo, marianismo, and negative cognitive-emotional factors: findings from the hispanic community health study/study of Latinos sociocultural ancillary study. *Journal of Latina/o Psychology,**4*(4), 202. 10.1037/lat0000050 10.1037/lat0000050 PMC5102330 27840779

[CR94] Nurullah, A. S. (2012). Received and provided social support: A review of current evidence and future directions. *American Journal of Health Studies,**27*, 173–188.

[CR95] Oken, M. M., Creech, R. H., Tormey, D. C., Horton, J., Davis, T. E., McFadden, E. T., & Carbone, P. P. (1982). Toxicity and response criteria of the Eastern Cooperative Oncology Group. *American Journal of Clinical Oncology,**5*, 649–656. 10.1097/00000421-198212000-00014 7165009

[CR96] Park, G. R., & Kim, J. (2021). Depressive symptoms among cancer patients: Variation by gender, cancer type, and social engagement. *Research in Nursing & Health,**44*, 811–821. 10.1002/nur.22168 34254692 10.1002/nur.22168

[CR97] Pasipanodya, E. C., Parrish, B. P., Laurenceau, J. P., Cohen, L. H., Siegel, S. D., Graber, E. C., & Belcher, A. J. (2012). Social constraints on disclosure predict daily well-being in couples coping with early-stage breast cancer. *Journal of Family Psychology,**26*, 661. 10.1037/a0028655 22686265 10.1037/a0028655

[CR98] Pedersen, A. F., Olesen, F., Hansen, R. P., Zachariae, R., & Vedsted, P. (2011). Social support, gender and patient delay. *British Journal of Cancer,**104*, 1249–1255. 10.1038/bjc.2011.87 21487428 10.1038/bjc.2011.87 PMC3078597

[CR99] Pedreira, P. B., Fleszar-Pavlović, S. E., Walsh, E. A., Noriega Esquives, B., Moreno, P. I., Perdomo, D., Heller, A. S., Antoni, M. H., & Penedo, F. J. (2024). Familism, family cohesion, and health-related quality of life in Hispanic prostate cancer survivors. *Journal of Behavioral Medicine,**2024*, 1–14. 10.1007/s10865-024-00479-1 10.1007/s10865-024-00479-1 PMC11293424 38429598

[CR100] Perez, G. K., Cruess, D. G., Cruess, S., Brewer, M., Stroop, J., Schwartz, R., & Greenstein, R. (2011). Attitudes toward direct-to-consumer advertisements and online genetic testing among high-risk women participating in a hereditary cancer clinic. *Journal of Health Communication,**16*, 607–628. 10.1080/10810730.2011.551993 21432710 10.1080/10810730.2011.551993

[CR101] Pfaendler, K. S., Wenzel, L., Mechanic, M. B., & Penner, K. R. (2015). Cervical cancer survivorship: Long-term quality of life and social support. *Clinical Therapeutics,**37*, 39–48. 10.1016/j.clinthera.2014.11.013 25592090 10.1016/j.clinthera.2014.11.013 PMC4404405

[CR102] Pilevarzadeh, M., Amirshahi, M., Afsargharehbagh, R., Rafiemanesh, H., Hashemi, S. M., & Balouchi, A. (2019). Global prevalence of depression among breast cancer patients: A systematic review and meta-analysis. *Breast Cancer Research and Treatment,**176*, 519–533. 10.1007/s10549-019-05271-3 31087199 10.1007/s10549-019-05271-3

[CR103] Pinquart, M., & Duberstein, P. (2010). Depression and cancer mortality: A meta-analysis. *Psychological Medicine,**40*, 1797–1810. 10.1017/s0033291709992285 20085667 10.1017/S0033291709992285 PMC2935927

[CR104] Polański, J., Chabowski, M., Świątoniowska-Lonc, N., Jankowska-Polańska, B., & Mazur, G. (2020). Can life satisfaction be considered a predictor of quality of life in patients with lung cancer? *European Review for Medical & Pharmacological Sciences,**24*, 11128–11138. 10.3390/nu13103332 33215430 10.26355/eurrev_202011_23600

[CR105] Porter, L. S., Keefe, F. J., Hurwitz, H., & Faber, M. (2005). Disclosure between patients with gastrointestinal cancer and their spouses. *Psycho-Oncology,**14*, 1030–1042. 10.1002/pon.915 15712247 10.1002/pon.915

[CR106] Powdthavee, N. (2008). Putting a price tag on friends, relatives, and neighbours: Using surveys of life satisfaction to value social relationships. *The Journal of Socio-Economics,**37*, 1459–1480. 10.1016/j.socec.2007.04.004

[CR107] Proto, E., & Rustichini, A. (2015). Life satisfaction, income, and personality. *Journal of Economic Psychology,**48*, 17–32. 10.1016/j.joep.2015.02.001

[CR108] Radloff, L. S. (1977). The CES-D scale: A self-report depression scale for research in the general population. *Applied Psychological Measurement,**1*, 385–401. 10.1177/014662167700100306

[CR109] Rini, C., Redd, W. H., Austin, J., Mosher, C. E., Meschian, Y. M., Isola, L., Scigliano, E., Moskowitz, C. H., Papadopoulos, E., Labay, L. E., & Rowley, S. (2011). Effectiveness of partner social support predicts enduring psychological distress after hematopoietic stem cell transplantation. *Journal of Consulting and Clinical Psychology,**79*, 64. 10.1037/a0022199 21261435 10.1037/a0022199 PMC3690958

[CR110] Rivera Rivera, J. N., & Burris, J. L. (2020). A systematic literature review and head-to-head comparison of social support and social constraint in relation to the psychological functioning of cancer survivors. *Annals of Behavioral Medicine,**54*, 176–192. 10.1093/abm/kaz037 31581293 10.1093/abm/kaz037 PMC7455805

[CR111] Rivera Rivera, J. N., Badour, C. L., & Burris, J. L. (2021). The association between psychological functioning and social support and social constraint after cancer diagnosis: A 30-day daily diary study. *Journal of Behavioral Medicine,**44*, 355–367. 10.1007/s10865-021-00200-6 33506286 10.1007/s10865-021-00200-6 PMC8131225

[CR112] Robin DiMatteo, M., Kelly, B., & Haskard‐Zolnierek,. (2010). Impact of depression on treatment adherence and survival from cancer. In D. W. Kissane, M. Maj, & N. Sartorius (Eds.), *Depression and cancer* (pp. 101–124). Wiley. 10.1002/9780470972533.ch5

[CR113] Ruiz-Rodríguez, I., Hombrados-Mendieta, I., Melguizo-Garín, A., & Martos-Méndez, M. J. (2022). The importance of social support, optimism, and resilience on the quality of life of cancer patients. *Frontiers in Psychology,**13*, 833176. 10.3389/fpsyg.2022.833176 35356348 10.3389/fpsyg.2022.833176 PMC8959607

[CR114] Salander, P., & Hamberg, K. (2005). Gender differences in patients’ written narratives about being diagnosed with cancer. *Psycho-Oncology,**14*, 684–695. 10.1002/pon.895 15669017 10.1002/pon.895

[CR115] Salonen, P., Tarkka, M. T., Kellokumpu-Lehtinen, P. L., Koivisto, A. M., Aalto, P., & Kaunonen, M. (2013). Effect of social support on changes in quality of life in early breast cancer patients: A longitudinal study. *Scandinavian Journal of Caring Sciences,**27*, 396–405. 10.1111/j.1471-6712.2012.01050.x 22834764 10.1111/j.1471-6712.2012.01050.x

[CR116] Sanson-Fisher, R., Girgis, A., Boyes, A., Bonevski, B., Burton, L., & Cook, P. (2000). The unmet supportive care needs of patients with cancer. *Cancer,**88*, 226–237. 10.1002/(SICI)1097-0142(20000101)88:1%3c226::AID-CNCR30%3e3.0.CO;2-P 10618627 10.1002/(sici)1097-0142(20000101)88:1<226::aid-cncr30>3.3.co;2-g

[CR117] Satin, J. R., Linden, W., & Phillips, M. J. (2009). Depression as a predictor of disease progression and mortality in cancer patients: A meta-analysis. *Cancer,**115*, 5349–5361. 10.1002/cncr.24561 19753617 10.1002/cncr.24561

[CR118] Schmidt, J. E., & Andrykowski, M. A. (2004). The role of social and dispositional variables associated with emotional processing in adjustment to breast cancer: An internet-based study. *Health Psychology,**23*, 259. 10.1037/0278-6133.23.3.259 15099166 10.1037/0278-6133.23.3.259

[CR154] Schroevers, M. J., Helgeson, V. S., Sanderman, R., & Ranchor, A. V. (2010). Type of social support matters for prediction of posttraumatic growth among cancer survivors. *Psycho-Oncology,**19*(1), 46–53. 10.1002/pon.1501 10.1002/pon.1501 19253269

[CR119] Sharpley, C. F., Bitsika, V., & Christie, D. H. (2013). Are somatic symptoms a legitimate part of the depression profile in prostate cancer patients? *Oncology Research and Treatment,**36*, 110–114. 10.1159/000348531 10.1159/000348531 23485998

[CR120] Shim, E. J., Mehnert, A., Koyama, A., Cho, S. J., Inui, H., Paik, N. S., & Koch, U. (2006). Health-related quality of life in breast cancer: A cross-cultural survey of German, Japanese, and South Korean patients. *Breast Cancer Research and Treatment,**99*, 341–350. 10.1007/s10549-006-9216-x 16685589 10.1007/s10549-006-9216-x

[CR121] Sippel, L. M., Pietrzak, R. H., Charney, D. S., Mayes, L. C., & Southwick, S. M. (2015). How does social support enhance resilience in the trauma-exposed individual? *Ecology and Society,**20*, 10. 10.5751/es-07832-200410

[CR122] Smith, H. R. (2015). Depression in cancer patients: Pathogenesis, implications and treatment. *Oncology Letters,**9*, 1509–1514. 10.3892/ol.2015.2944 25788991 10.3892/ol.2015.2944 PMC4356432

[CR123] Soriano, E. C., Otto, A. K., LoSavio, S. T., Perndorfer, C., Siegel, S. D., & Laurenceau, J. P. (2021). Fear of cancer recurrence and inhibited disclosure: Testing the social-cognitive processing model in couples coping with breast cancer. *Annals of Behavioral Medicine,**55*, 192–202. 10.1093/abm/kaaa043 32608472 10.1093/abm/kaaa043 PMC7980765

[CR124] Soriano, E. C., Otto, A. K., Siegel, S. D., & Laurenceau, J. P. (2017). Partner social constraints and early-stage breast cancer: Longitudinal associations with psychosexual adjustment. *Journal of Family Psychology,**31*, 574. 10.1037/fam0000302 28206777 10.1037/fam0000302 PMC5555802

[CR153] Sprecher, S., & Hendrick, S. S. (2004). Self-disclosure in intimate relationships: Associations with individual and relationship characteristics over time. *Journal of Social and Clinical Psychology,**23*(6), 857–877. 10.1521/jscp.23.6.857.54803

[CR125] Stephens, C., Stein, K., & Landrine, H. (2010). The role of acculturation in life satisfaction among Hispanic cancer survivors: Results of the American Cancer Society’s study of cancer survivors. *Psycho-Oncology,**19*, 376–383. 10.1002/pon.1566 19367560 10.1002/pon.1566

[CR126] Swartzman, S., Sani, F., & Munro, A. J. (2017). The role of social support, family identification, and family constraints in predicting posttraumatic stress after cancer. *Psycho-Oncology,**26*, 1330–1335. 10.1002/pon.4304 27862598 10.1002/pon.4304

[CR127] Teasdale, J. D. (1983). Negative thinking in depression: Cause, effect, or reciprocal relationship? *Advances in Behaviour Research and Therapy,**5*, 3–25. 10.1016/0146-6402(83)90013-9

[CR128] Theofilou, P., & Panagiotaki, H. (2012). A literature review to investigate the link between psychosocial characteristics and treatment adherence in cancer patients. *Oncology Reviews,**6*(1), e5. 10.4081/oncol.2012.178 25992207 10.4081/oncol.2012.e5 PMC4419635

[CR129] Thompson, T., Pérez, M., Kreuter, M., Margenthaler, J., Colditz, G., & Jeffe, D. B. (2017). Perceived social support in African American breast cancer patients: Predictors and effects. *Social Science & Medicine,**192*, 134–142. 10.1016/j.socscimed.2017.09.035 28965004 10.1016/j.socscimed.2017.09.035 PMC5693247

[CR130] Tsai, T. C., Lee, G. G., Ting, A., Antoni, M. H., Mendez, A., Carver, C. S., & Kim, Y. (2023). Roles of benefit finding in psychological and inflammatory adjustments in persons with colorectal cancer: A prospective analysis on the multidimensionality of benefit finding. *Psychology & Health,**2023*, 1–19. 10.1080/08870446.2023.2238280 10.1080/08870446.2023.2238280 PMC10805970 37488833

[CR131] Tyson, D. M., Vázquez-Otero, C., Medina-Ramirez, P., Arriola, N. B., McMillan, S. C., & Gwede, C. K. (2017). Understanding the supportive care needs of Hispanic men cancer survivors. *Ethnicity & Health,**22*, 1–16. 10.1080/13557858.2016.1196649 27350450 10.1080/13557858.2016.1196649 PMC5351415

[CR132] Uchino, B. N. (2009). Understanding the links between social support and physical health: A life-span perspective with emphasis on the separability of perceived and received support. *Perspectives on Psychological Science,**4*, 236–255. 10.1111/j.1745-6924.2009.01122.x 26158961 10.1111/j.1745-6924.2009.01122.x

[CR133] Vázquez, C., Rahona, J. J., Gomez, D., Caballero, F. F., & Hervas, G. (2015). A national representative study of the relative impact of physical and psychological problems on life satisfaction. *Journal of Happiness Studies,**16*, 135–148. 10.1007/s10902-014-9501-z

[CR134] Walker, Z. J., Xue, S., Jones, M. P., & Ravindran, A. V. (2021). Depression, anxiety, and other mental disorders in patients with cancer in low-and lower-middle–income countries: A systematic review and meta-analysis. *JCO Global Oncology,**7*, 1233–1250. 10.1200/go.21.00056 34343029 10.1200/GO.21.00056 PMC8457869

[CR135] Wang, L., Wilson, S. E., Stewart, D. B., & Hollenbeak, C. S. (2011). Marital status and colon cancer outcomes in US Surveillance, Epidemiology and End Results registries: Does marriage affect cancer survival by gender and stage? *Cancer Epidemiology,**35*, 417–422. 10.1016/j.canep.2011.02.004 21466984 10.1016/j.canep.2011.02.004

[CR136] Wang, X., Wang, N., Zhong, L., Wang, S., Zheng, Y., Yang, B., Zhang, J., Lin, Y., & Wang, Z. (2020). Prognostic value of depression and anxiety on breast cancer recurrence and mortality: A systematic review and meta-analysis of 282,203 patients. *Molecular Psychiatry,**25*, 3186–3197. 10.1038/s41380-020-00865-6 32820237 10.1038/s41380-020-00865-6 PMC7714689

[CR137] Watts, S., Leydon, G., Birch, B., Prescott, P., Lai, L., Eardley, S., & Lewith, G. (2014). Depression and anxiety in prostate cancer: A systematic review and meta-analysis of prevalence rates. *British Medical Journal Open,**4*, e003901. 10.1136/bmjopen-2013-003901 10.1136/bmjopen-2013-003901 PMC3963074 24625637

[CR138] Wellisch, D., Kagawa-Singer, M., Reid, S. L., Lin, Y. J., Nishikawa-Lee, S., & Wellisch, M. (1999). An exploratory study of social support: A cross-cultural comparison of Chinese-, Japanese-, and Anglo-American breast cancer patients. *Psycho-Oncology,**8*(3), 207–219. 10.1002/(SICI)1099-1611(199905/06)8:3%3c207::AID-PON357%3e3.0.CO;2-B 10390733 10.1002/(SICI)1099-1611(199905/06)8:3<207::AID-PON357>3.0.CO;2-B

[CR139] Wen, S., Xiao, H., & Yang, Y. (2019). The risk factors for depression in cancer patients undergoing chemotherapy: A systematic review. *Supportive Care in Cancer,**27*, 57–67. 10.1007/s00520-018-4466-9 30225571 10.1007/s00520-018-4466-9

[CR140] Widows, M. R., Jacobsen, P. B., & Fields, K. K. (2000). Relation of psychological vulnerability factors to posttraumatic stress disorder symptomatology in bone marrow transplant recipients. *Psychosomatic Medicine,**62*, 873–882. 10.1097/00006842-200011000-00018 11139008 10.1097/00006842-200011000-00018

[CR141] Wingard, J. R., Huang, I. C., Sobocinski, K. A., Andrykowski, M. A., Cella, D., Rizzo, J. D., Brady, M., Horowitz, M. M., & Bishop, M. M. (2010). Factors associated with self-reported physical and mental health after hematopoietic cell transplantation. *Biology of Blood and Marrow Transplantation,**16*, 1682–1692. 10.1016/j.bbmt.2010.05.017 20685400 10.1016/j.bbmt.2010.05.017 PMC2975785

[CR142] Wong, C. C., Warmoth, K., Ivy, S., Cheung, B., & Lu, Q. (2018). Relation of social constraints on disclosure to adjustment among Chinese American cancer survivors: A multiprocess approach. *Psycho-Oncology,**27*(3), 977–982. 10.1002/pon.4604 29232487 10.1002/pon.4604

[CR156] Yanez, B., McGinty, H. L., Buitrago, D., Ramirez, A. G., & Penedo, F. J. (2016). Cancer outcomes in Hispanics/Latinos in the United States: An integrative review and conceptual model of determinants of health. *Journal of Latina/o Psychology,**4*(2), 114. 10.1037/lat0000055 10.1037/lat0000055 PMC4943845 27429867

[CR143] Zakowski, S. G., Harris, C., Krueger, N., Laubmeier, K. K., Garrett, S., Flanigan, R., & Johnson, P. (2003). Social barriers to emotional expression and their relations to distress in male and female cancer patients. *British Journal of Health Psychology,**8*, 271–286. 10.1348/135910703322370851 14606973 10.1348/135910703322370851

[CR144] Zhang, S., Yang, Z., Qiu, P., Li, J., & Zhou, C. (2022). Research on the role of marriage status among women who underwent breast reconstruction following mastectomy: A competing risk analysis model based on the SEER database, 1998–2015. *Frontiers in Surgery,**8*, 803223. 10.3389/fsurg.2021.803223 35127805 10.3389/fsurg.2021.803223 PMC8814322

[CR145] Zhou, J., Chen, X., Wang, Z., & Li, Q. (2023a). Couple-based communication interventions for cancer patient–spousal caregiver dyads’ psychosocial adaptation to cancer: A systematic review. *Healthcare,**11*, 236. 10.3390/healthcare11020236 36673604 10.3390/healthcare11020236 PMC9858755

[CR146] Zhou, Y., Che, C. C., Chong, M. C., Zhao, H., & Lu, Y. (2023b). Effects of marital self-disclosure on marital relationship and psychological outcome for cancer patients: A systematic review. *Supportive Care in Cancer,**31*, 361. 10.1007/s00520-023-07826-z 37249639 10.1007/s00520-023-07826-z

